# Comprehensive analysis of the ischemic stroke burden at global, regional, and national levels (1990–2021): trends, influencing factors, and future projections

**DOI:** 10.3389/fneur.2025.1492691

**Published:** 2025-03-19

**Authors:** Haonan Zhao, Sikai Lu, Yang Jie, Wu Chao, Wenxia Zhu, Dongya Huang

**Affiliations:** ^1^Department of Neurology, Shanghai East Hospital, Tongji University School of Medicine, Shanghai, China; ^2^Pudong New Area Sanlin Community Health Service Center, Shanghai, China

**Keywords:** global burden of disease study, ischemic stroke, Joinpoint regression, trends analysis, preventive health services, forecasting analysis

## Abstract

**Background and purpose:**

Estimating the global burden of ischemic strokes (IS) is crucial for enhancing prevention and control strategies.

**Methods:**

We collected four epidemiological indicators—prevalence, incidence, deaths, and disability-adjusted life years (DALYs)—for ischemic stroke (IS) from the Global Burden of Disease (GBD) database, which covers the years 1990 to 2021. Our research analyzed the features of the IS burden and described the trends of these four indicators.

**Results:**

The Joinpoint and age-period-cohort models reflected the changing trends in age-standardized indicators. Decomposition analysis examined the factors influencing each epidemiological indicator. The Bayesian Age-Period-Cohort (BAPC) model detailed changes in the number and rate of IS from 1990 to 2021 and projected trends through 2046. The Norpred model was used to verify the stability of the BAPC prediction results. The prevalence, incidence, deaths, and DALYs due to IS generally exhibited a downward trend. However, the predictions indicated that while the age-standardized incidence rate decreased from 1990 to 2015, this trend reversed between 2016 and 2021 and is expected to continue until 2046. This reversal is likely driven by factors such as population aging, given that age is a strongly correlated risk factor for IS. The IS burden was negatively associated with socio-demographic index (SDI) levels, with high systolic blood pressure identified as the largest risk factor for DALYs and deaths. The consistency between the BAPC and Norpred models enhances the reliability of these projections.

**Conclusion:**

Over the past two decades, trends in prevalence, incidence, deaths, and DALYs have all declined. However, projections suggest that incidence will show an upward trend over the next two decades.

## 1 Introduction

Stroke, classified into ischemic and hemorrhagic subtypes, remains the second leading cause of death worldwide and the primary cause of disability both globally and in China, imposing a significant economic burden on society and resulting in poor quality of life for patients (1–3). Ischemic stroke (IS), which accounts for approximately 87% of stroke subtypes, is defined as an IS resulting from blockage of cerebral arteries. The restoration of blood flow as soon as possible through intravenous thrombolysis or mechanical thrombolysis is considered to be the primary treatment for IS (4, 5).

Nevertheless, the elevated mortality and disability rates observed in IS are attributable to several factors, including the limited duration of treatment windows and reperfusion injury resulting from oxidative stress and inflammation (6, 7). In addition to effective treatment, primary and secondary preventive measures, such as lifestyle modifications and blood pressure management, are crucial for the prevention of IS (8). Understanding the changing epidemiology of IS is significantly important for both clinical practice and public health to enhance stroke outcomes.

The Global Burden of Disease (GBD) Study aims to quantify the financial burden of health in diverse geographical regions over time, enhancing the efficacy of health systems and eliminating disparities^1^ (9, 10). The GBD collected data on 369 diseases and injuries across 204 countries, providing a comprehensive and annually updated assessment of the occurrence, prevalence, and mortality rates of disease and injury (10, 11). This offers a valuable opportunity to synthesize the distribution of global pressures for IS.

In this study, we analyzed the incidence, prevalence, mortality, and disability-adjusted life years (DALYs) of IS from 1990 to 2021, along with potential trends from 1990 to 2046 based on GBD 2021. This analysis establishes a foundation for enhancing primary and secondary prevention efforts and improving prognosis.

## 2 Methods

### 2.1 Data source

The data on the burden of IS in the present research were obtained from the GBD 2021, which provides researchers with easily accessible, integrated, and comprehensive epidemiological data. The GBD 2021 aggregates seven epidemiological indicators (such as death, incidence, prevalence, years of loss, years of life lost due to disability, DALYs, and maternal mortality ratio) for 376 diseases and injuries across 204 countries and territories, as well as 88 risk factors. Our research queried IS data through the Institute for Health Metrics and Evaluation (IHME). We selected “ischemic stroke” as the cause, four epidemiological indicators (prevalence, incidence, death, and DALYs) as measures, and three expressed units (number, percent, and rate). Based on socio-demographic index (SDI), gender, and age groups, we further analyzed and described the retrieved data. IS is defined as any atherosclerotic or thromboembolic event (excluding transient ischemic episodes) that obstructs blood flow to brain tissue and leads to the development of a cerebral infarction (12).

### 2.2 Statistical description and analysis

The prevalence, incidence, deaths, and DALYs reflect the epidemiological trend of IS. To more accurately assess the disease burden of IS, age-standard rates (ASR) of prevalence (ASPR), incidence (ASIR), deaths (ASMR), and DALYs (ASDR) were calculated, reducing the heterogeneity caused by differences in the age structure of the population. Furthermore, to reflect the changing trend of the age-standard epidemiological indicators from 1990 to 2021, estimated annual percentage change (EAPC) and annual average percentage change (AAPC) were calculated. Previous research has detailed the concept and formula of EAPC (13, 14). Joinpoint regression software (version 4.9.1.0) analyzed the changes in the epidemiological trend, identified points of significant change, and calculated the annual percentage change (APC) and AAPC. Conventional descriptive methods are limited to describing only changes in epidemiological indicators over time and cannot eliminate or control for interactions between age, period, and cohort. In contrast, the age-period-cohort model integrates these three factors to reflect trends in disease changes by age, period, and cohort (15). The SDI is a composite indicator combining per capita income, average total education, and fertility rate to evaluate a country’s development status. We also analyzed the relationship between the five-level SDI and the burden of IS. The population attributable fraction (PAF) was used to estimate risk factors associated with changes in ASMR and DALY. A decomposition analysis of the incidence of IS was conducted to ascertain how much population-level changes have contributed to variations in incidence over the past 32 years. The Bayesian Age-Period-Cohort (BAPC) model integrates age, period, and cohort effects to describe the number and rate of changes in IS from 1990 to 2021 and to project trends to 2046. To verify the stability of the BAPC prediction results, a Norpred model was performed (16, 17).

## 3 Result

### 3.1 The distribution of IS in 2021

**Figure 1:**
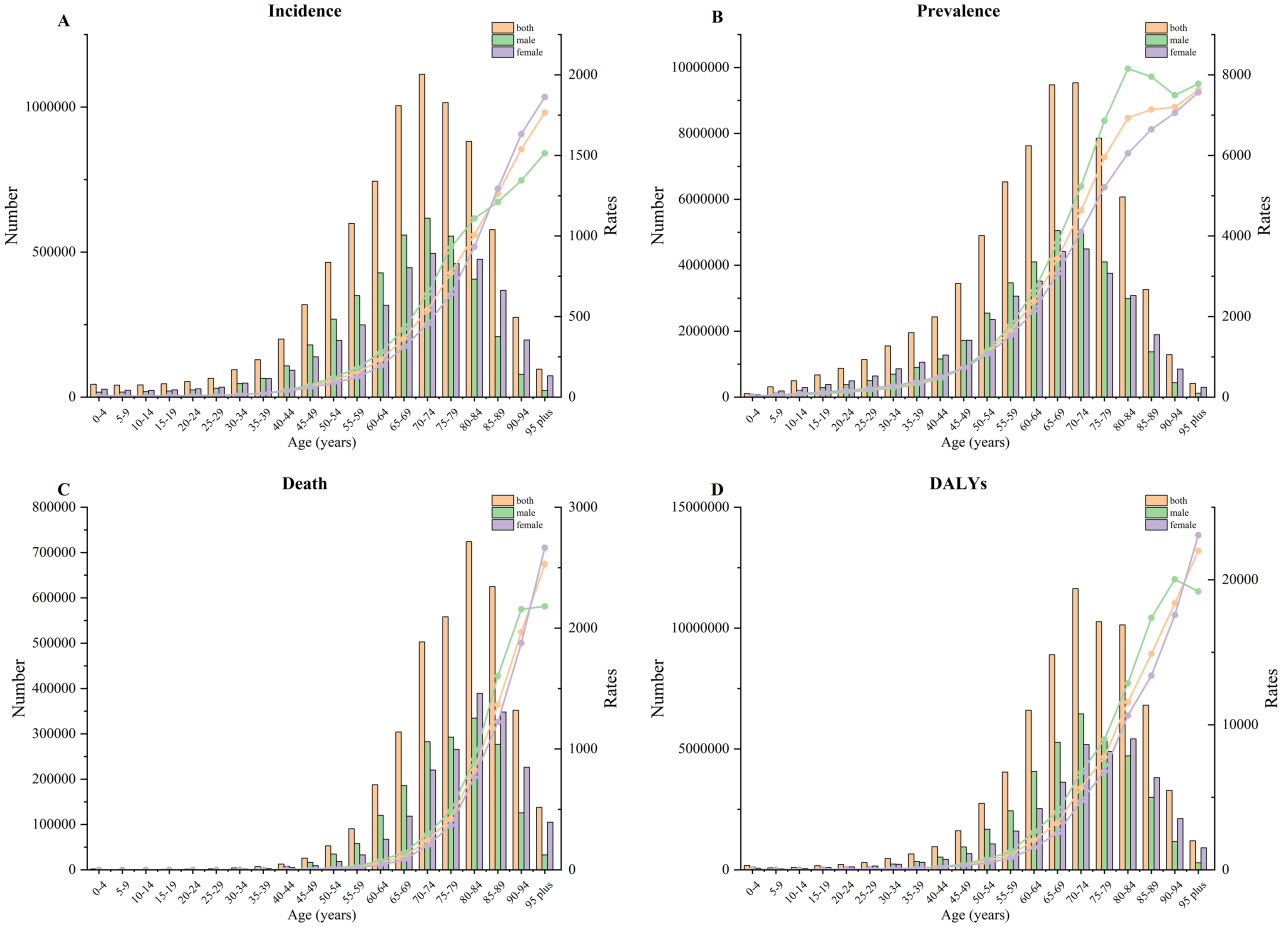
The change trend of incidence **(A)**, prevalence **(B)**, death **(C)**, and DALYs **(D)** by age in 2021.

The distribution of incidence, prevalence, deaths, and DALYs across different age groups displayed a near-unimodal pattern in cases of IS (Figure 1). The highest incidence, prevalence, and DALYs of IS were found in the 70–74 years age group. IS deaths peaked in the 80–84 years age group. Prevalence was greater in men than in women across all age groups. However, deaths and DALYs related to IS were higher in men than in women before reaching 90 years, while the opposite was observed after 90. The incidence of IS was also more prevalent in men than in women below the age of 85, with the reverse seen above this cutoff. Additionally, age-standardized rates (ASR) of incidence, prevalence, deaths, and DALYs due to IS exhibited an overall upward trend with age.

### 3.2 Global trend of IS from 1990 to 2021

**Figure 2:**
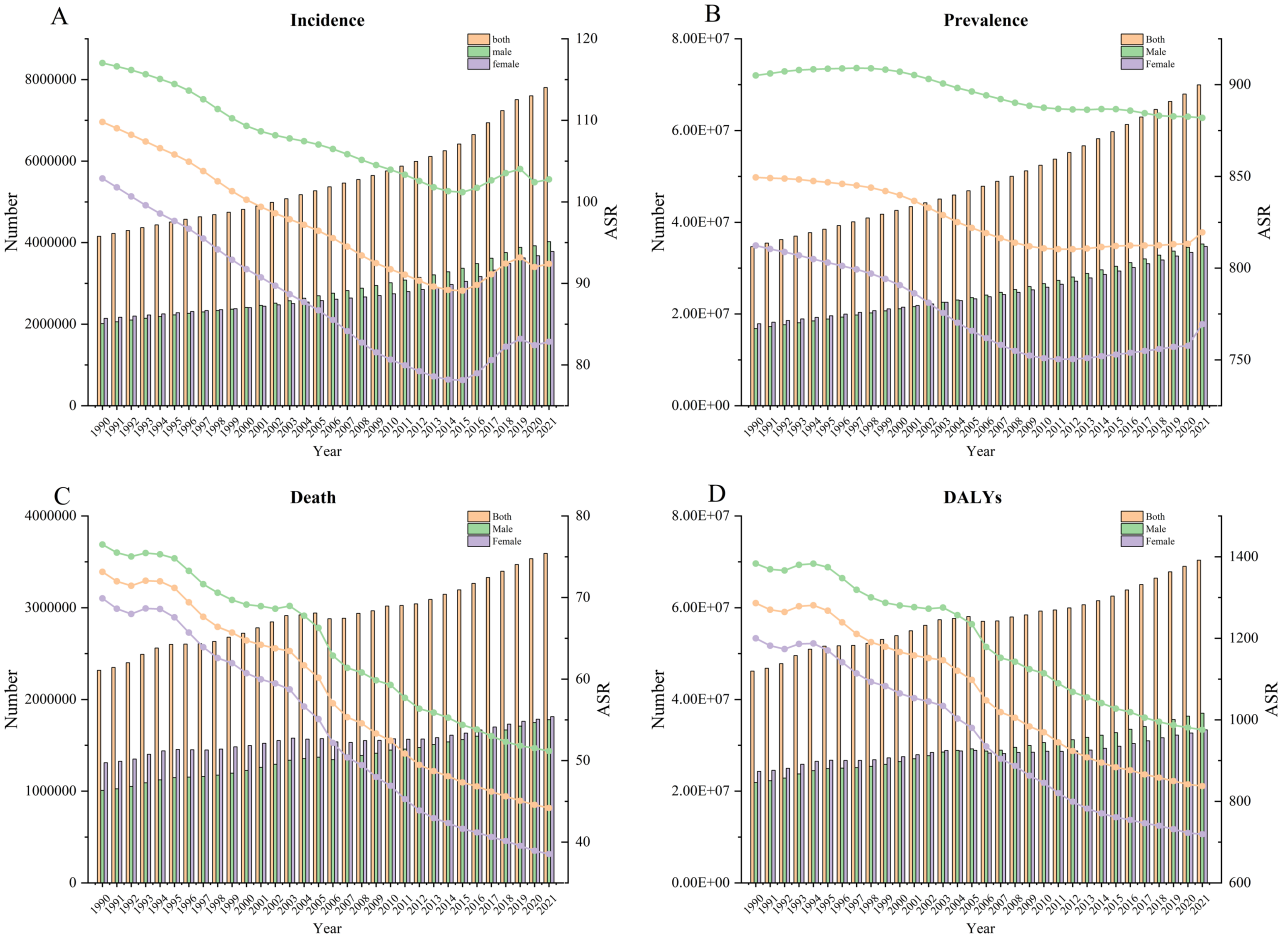
The change trend of incidence **(A)**, prevalence **(B)**, death **(C)**, and DALYs **(D)** from 1990 to 2021.

Overall, the ASIR of IS exhibited a decline from 109.79 (93.56 to 127.62) in 1990 to 92.39 (79.84 to 105.82) in 2019, representing a 15.85% reduction (Table 1). Similarly, the ASMR (EAPC -1.83, 95% CI: −1.92 to −1.74), ASPR (EAPC -0.67, 95% CI: −0.76 to −0.58), and DALYs rate (EAPC -1.59, 95% CI: −1.68 to −1.5) of IS also decreased significantly (Figure 2; Supplementary Tables S1–S3). APC by Joinpoint regression analysis is shown in Table 2. From 1990 to 2021, there was a general downward trend in ASIR in IS (AAPC = −0.57, *p* < 0.05). However, this trend reversed in 2015, with ASIR increasing significantly from 2015 to 2019 (APC = 1.17, *p* < 0.05) and then decreasing slightly after 2020 (APC = −0.58, *p* < 0.05). A similar pattern was observed in the ASPR, ASMR, and DALYs, which also demonstrated a general downward trend. The ASPR exhibited the most pronounced decline from 1990 to 2006 (APC = −0.42, *p* < 0.05), followed by the ASMR (APC = −3.21, *p* < 0.05) and DALYs (APC = −3.31, *p* < 0.05). This decline was most notable between 2003 and 2007.

**Table 1:** Numbers and ASR per 100,000 cases of IS incidence in 1990 and 2021, along with the relative changes and EAPC in ASR per 100,000 cases from 1990 to 2021, categorized by global, SDI, and GBD regions.

Characteristic	Number in 1990 (95% CI)	Age-standardized rate in 1990 (95% CI)	Number in 2021 (95% CI)	Age-standardized rate in 2021 (95% CI)	Relative change of numbers from 1990 to 2021	Relative change of age-standardized rate from 1990 to 2021	EAPC (age-standardized rate, 95% CI)
Andean Latin America	13,563 (11,785 to 15,387)	63.46 (55.42 to 71.89)	27,415 (24,042 to 31,116)	46.29 (40.6 to 52.41)	102.14%	−27.05%	−1.13 (−1.24 to −1.02)
Australasia	20,989 (19,362 to 22,541)	91.09 (84.12 to 97.82)	28,160 (25,188 to 31,109)	52.76 (47.35 to 58.52)	34.16%	−42.08%	−2.05 (−2.2 to −1.91)
Caribbean	19,577 (17,216 to 21,953)	76.19 (67.54 to 85.71)	36,042 (32,142 to 39,961)	67.34 (59.96 to 74.74)	84.10%	−11.61%	−0.42 (−0.46 to −0.37)
Central Asia	65,959 (58,344 to 73,949)	141.86 (124.6 to 159.13)	102,573 (91,018 to 114,463)	132.92 (118.17 to 148.1)	55.51%	−6.30%	−0.2 (−0.29 to −0.11)
Central Europe	222,510 (193,305 to 251,309)	157.98 (138.35 to 177.71)	238,905 (209,922 to 266,604)	107 (95.27 to 118.4)	7.37%	−32.27%	−1.35 (−1.4 to −1.3)
Central Latin America	68,643 (58,879 to 78,851)	80.45 (69.05 to 93.22)	127,075 (110,155 to 144,196)	51.96 (45.09 to 58.83)	85.12%	−35.42%	−1.67 (−1.82 to −1.51)
Central Sub-Saharan Africa	27,144 (23,222 to 32,005)	122.67 (104.25 to 142.58)	59,173 (51,496 to 68,041)	108.99 (94.95 to 124.84)	117.99%	−11.15%	−0.41 (−0.45 to −0.38)
East Asia	800,330 (656,281 to 982,073)	101.31 (82.99 to 121.88)	2,850,090 (2,363,158 to 3,405,882)	134.77 (112.62 to 158.4)	256.11%	33.02%	0.86 (0.8 to 0.92)
Eastern Europe	515,846 (425,258 to 623,601)	197.9 (164.89 to 232.39)	490,197 (415,356 to 571,451)	142.57 (122.12 to 164.67)	−4.97%	−27.96%	−1.13 (−1.24 to −1.02)
Eastern Sub-Saharan Africa	85,162 (72,719 to 99,207)	110.88 (94.18 to 130.12)	179,318 (154,966 to 204,334)	103.55 (89.22 to 118.4)	110.56%	−6.61%	−0.21 (−0.26 to −0.17)
Global	4,151,978 (3,536,772 to 4,868,150)	109.79 (93.56 to 127.62)	7,804,449 (6,719,760 to 8,943,692)	92.39 (79.84 to 105.82)	87.97%	−15.85%	−0.67 (−0.76 to −0.58)
High SDI	1,153,374 (992,806 to 1,333,283)	105.54 (91.16 to 121.17)	1,351,913 (1,179,761 to 1,535,710)	66.05 (58.24 to 74.72)	17.21%	−37.41%	−1.75 (−1.86 to −1.64)
High-income Asia Pacific	234,946 (193,848 to 280,991)	120.98 (100.7 to 143.98)	284,445 (249,283 to 322,678)	64.59 (56.36 to 73.72)	21.07%	−46.61%	−2.53 (−2.73 to −2.33)
High-income North America	315,394 (259,208 to 383,545)	89.95 (74.41 to 108.35)	352,963 (299,100 to 413,294)	56.86 (48.63 to 66.11)	11.91%	−36.79%	−1.58 (−1.73 to −1.43)
High-middle SDI	1,306,277 (1,096,265 to 1,547,833)	141.37 (119.89 to 164.14)	2,243,340 (1,899,825 to 2,614,463)	116.04 (99.23 to 133.86)	71.74%	−17.92%	−0.74 (−0.85 to −0.63)
Low SDI	216,493 (183,974 to 252,019)	94.39 (80.38 to 110.81)	433,298 (374,565 to 494,218)	82.17 (71.04 to 93.1)	100.14%	−12.94%	−0.51 (−0.56 to −0.46)
Low-middle SDI	516,851 (439,586 to 603,885)	87.34 (74.13 to 102.39)	1,120,427 (971,630 to 1,273,722)	80.66 (69.98 to 91.67)	116.78%	−7.65%	−0.34 (−0.38 to −0.3)
Middle SDI	953,541 (800,912 to 1,134,827)	97.51 (81.45 to 115.65)	2,648,525 (2,240,569 to 3,082,587)	103.51 (88.01 to 119.98)	177.76%	6.16%	0.12 (0.07 to 0.16)
North Africa and the Middle East	199,912 (175,528 to 229,003)	117.53 (102.61 to 133.52)	461,146 (409,815 to 516,837)	103.58 (91.57 to 116.06)	130.67%	−11.87%	−0.43 (−0.47 to −0.39)
Oceania	2,343 (2017 to 2,728)	81.5 (70.05 to 94.27)	5,311 (4,625 to 6,003)	73.2 (63.72 to 82.91)	126.70%	−10.18%	−0.43 (−0.47 to −0.39)
South Asia	398,076 (331,672 to 474,038)	72.63 (60.6 to 86.45)	853,370 (727,638 to 990,209)	61.16 (52.33 to 70.75)	114.37%	−15.78%	−0.74 (−0.82 to −0.66)
Southeast Asia	265,779 (228,020 to 310,151)	107.32 (91.5 to 125.56)	667,263 (582,281 to 763,035)	107.31 (93.68 to 122.69)	151.06%	−0.01%	−0.04 (−0.07 to 0)
Southern Latin America	44,172 (38,720 to 49,910)	97.84 (86.31 to 109.85)	52,070 (46,169 to 58,185)	60.35 (53.46 to 67.61)	17.88%	−38.32%	−1.77 (−1.93 to −1.62)
Southern Sub-Saharan Africa	33,295 (28,141 to 39,225)	122.29 (101.93 to 146.66)	64,729 (54,456 to 74,991)	121.86 (102.46 to 142.18)	94.41%	−0.35%	−0.11 (−0.36 to 0.15)
Tropical Latin America	106,000 (88,156 to 125,886)	119.16 (98.34 to 142.27)	166,749 (140,998 to 194,698)	66.42 (55.99 to 77.52)	57.31%	−44.26%	−2.04 (−2.16 to −1.92)
Western Europe	613,766 (538,423 to 692,798)	105.97 (93.99 to 117.98)	549,191 (496,802 to 601,606)	58.14 (52.88 to 63.67)	−10.52%	−45.13%	−2.12 (−2.21 to −2.03)
Western Sub-Saharan Africa	98,570 (83,977 to 114,607)	104.07 (88.24 to 122.05)	208,265 (181,391 to 236,454)	94.74 (82.14 to 106.92)	111.29%	−8.96%	−0.29 (−0.33 to −0.25)

**Table 2:** Joinpoint regression analysis of IS, ASIR, ASPR, ASMR, and DALYs globally from 1990 to 2021.

	ASIR		ASPR		ASMR		DALYs	
	Period	APC	Period	APC	Period	APC	Period	APC
Both	**1990–2021 (AAPC)**	−0.57* (−0.65 to − 0.48)	**1990–2021 (AAPC)**	−0.12* (−0.14 to − 0.11)	**1990–2021 (AAPC)**	−1.60* (−1.39 to − 14.99)	**1990–2021 (AAPC)**	−1.37* (−1.2 to − 16.28)
	1990–1996	−0.75* (−0.85 to −0.65)	1990–1999	−0.09* (−0.1 to −0.08)	1990–21,995	−0.38 (0 to −2.11)	1990–1995	−0.13 (0.14 to −1.05)
	1999–2012	−0.89* (−0.93 to −0.86)	2006–2010	−0.26* (−0.31 to −0.21)	1998–2003	−0.80* (−0.31 to −3.49)	1998–2004	−0.85* (−0.61 to −7.37)
	2012–2015	−0.48 (−1.02 to 0.06)	2010–2019	0.04* (0.02 to 0.05)	2003–2007	−3.21* (−2.46 to −8.94)	2004–2007	−3.31* (−2.25 to −6.6)
	2015–2019	1.17* (0.9 to 1.44)	2019–2021	0.33* (0.2 to 0.46)	2007–2013	−2.24* (−1.87 to −12.69)	2007–2014	−1.90* (−1.69 to −19.31)
	2019–2021	−0.58 (−1.17 to 0)			2013–2021	−1.24* (−1.02 to −12.39)	2014–2021	−0.95* (−0.76 to −10.73)
Male	**1990–2021 (AAPC)**	−0.42* (−0.5 to −0.35)	**1990–2021 (AAPC)**	−0.09* (−0.1 to −0.07)	**1990–2021 (AAPC)**	−1.28* (−1.08 to −12.89)	**1990–2021 (AAPC)**	−1.11* (−0.96 to −14.32)
	1990–1995	−0.42* (−0.57 to −0.27)	1990–1993	0.12* (0.06 to 0.18)	1990–1995	−0.38 (0.01 to −2.08)	1990–1995	0.01 (0.3 to 0.08)
	1995–2000	−0.96* (−1.16 to −0.77)	1993–1999	0.01 (−0.01 to 0.04)	1995–1999	−1.79* (−1.01 to −4.84)	1995–1998	−2.03* (−0.93 to −3.91)
	2000–2006	−0.42* (−0.57 to −0.28)	1999–2009	−0.23* (−0.24 to −0.22)	1999–2004	−0.37 (0.06 to −1.83)	1998–2004	−0.41* (−0.18 to −3.84)
	2006–2015	−0.60* (−0.67 to −0.54)	2009–2012	−0.06 (−0.16 to 0.03)	2004–2007	−3.40* (−2.09 to −5.48)	2004–2007	−2.92* (−1.98 to −6.59)
	2015–2018	0.95* (0.36 to 1.55)	2012–2015	0 (−0.09 to 0.1)	2007–2017	−1.51* (−1.37 to −22.33)	2007–2015	−1.47* (−1.32 to −20.99)
	2018–2021	−0.35* (−0.66 to −0.04)	2015–2021	−0.10* (−0.11 to −0.08)	2017–2021	−0.82* (−0.19 to −2.77)	2015–2021	−0.91* (−0.68 to −8.39)
Female	**1990–2021 (AAPC)**	−0.72* (−0.78 to −0.65)	**1990–2021 (AAPC)**	−0.19* (−0.2 to −0.17)	**1990–2021 (AAPC)**	−1.88* (−1.66 to −16.43)	**1990–2021 (AAPC)**	−1.63* (−1.44 to −16.4)
	1990–2005	−1.15* (−1.18 to −1.13)	1990–1999	−0.24* (−0.25 to −0.23)	1990–1995	−0.49* (−0.12 to −2.81)	1990–1995	−0.32 (0.01 to −2.06)
	2005–2010	−1.51* (−1.69 to −1.32)	1999–2006	−0.62* (−0.64 to −0.6)	1995–1998	−2.67* (−1.03 to −3.46)	1995–1998	−2.43* (−1.04 to −3.7)
	2010–2015	−0.64* (−0.82 to −0.47)	2006–2010	−0.38* (−0.44 to −0.33)	1998–2003	−1.19* (−0.67 to −4.81)	1998–2003	−1.02* (−0.57 to −4.86)
	2015–2019	1.70* (1.41 to 1.98)	2010–2019	0.09* (0.08 to 0.1)	2003–2007	−3.65* (−2.82 to −9.25)	2003–2007	−3.22* (−2.49 to −9.28)
	2019–2021	−0.41 (−1.01 to 0.2)	2019–2021	0.74* (0.59 to 0.88)	2007–2013	−2.73* (−2.29 to −13.17)	2007–2013	−2.52* (−2.13 to −13.61)
					2013–2021	−1.35* (−1.11 to −11.81)	2013–2021	−1.05* (−0.83 to −9.98)

AAPC: Annual average percentage change. *p < 0.05.

### 3.3 Age, period, and cohort effects

Table 3 demonstrates the age-period-cohort effect on IS ASIR, ASPR, ASMR, and DALY rates. The ASIR showed an increase starting from the age range of 10–14 years, peaking in the age range of 90–94 years. The ASPR rose from the ages of 0–4 years, reaching its peak at the ages of 80–84 years. The ASMR and DALYs also increased from the ages of 15–19, peaking at ages 95 and older. Additionally, the ASIR, ASPR, ASMR, and DALYs displayed an upward trend between the periods of 1992–1996 and 2017–2021. The birth cohort data for each age group indicated that the ASIR, ASPR, ASMR, and DALYs of IS in the earlier period were lower than those in the later period across all age groups.

**Table 3:** Age-period-cohort effects of IS incidence, prevalence, mortality, and DALY rates globally.

Factor	Incidence		Prevalence		Death		DALYs	
	RR	95%CI	RR	95%CI	RR	95%CI	RR	95%CI
Age (years)								
0–4	0.240	0.239 to 0.241	0.064	0.063 to 0.064	0.255	0.251 to 0.26	0.285	0.284 to 0.285
5–9	0.180	0.179 to 0.181	0.159	0.159 to 0.159	0.037	0.036 to 0.038	0.076	0.075 to 0.076
10–14	0.166	0.165 to 0.166	0.229	0.229 to 0.229	0.021	0.02 to 0.022	0.074	0.074 to 0.074
15–19	0.175	0.174 to 0.175	0.292	0.292 to 0.292	0.053	0.051 to 0.054	0.12	0.119 to 0.12
20–24	0.192	0.192 to 0.193	0.352	0.352 to 0.352	0.065	0.063 to 0.066	0.145	0.144 to 0.145
25–29	0.214	0.213 to 0.214	0.414	0.413 to 0.414	0.08	0.078 to 0.081	0.173	0.173 to 0.173
30–34	0.271	0.271 to 0.272	0.483	0.483 to 0.484	0.122	0.121 to 0.124	0.228	0.228 to 0.229
35–39	0.353	0.353 to 0.354	0.58	0.579 to 0.58	0.192	0.189 to 0.194	0.31	0.31 to 0.31
40–44	0.572	0.571 to 0.573	0.724	0.723 to 0.724	0.335	0.332 to 0.339	0.461	0.461 to 0.462
45–49	0.885	0.884 to 0.887	0.984	0.983 to 0.984	0.621	0.615 to 0.626	0.744	0.744 to 0.745
50–54	1.239	1.237 to 1.24	1.349	1.349 to 1.35	1.116	1.107 to 1.124	1.191	1.19 to 1.192
55–59	1.593	1.591 to 1.595	1.811	1.81 to 1.812	1.744	1.734 to 1.755	1.696	1.695 to 1.697
60–64	2.197	2.195 to 2.200	2.338	2.337 to 2.339	3.644	3.628 to 3.661	2.997	2.996 to 2.999
65–69	2.952	2.950 to 2.955	2.985	2.984 to 2.987	5.478	5.458 to 5.498	3.956	3.954 to 3.958
70–74	3.866	3.862 to 3.869	3.613	3.611 to 3.615	10.073	10.041 to 10.105	6.089	6.086 to 6.091
75–79	4.799	4.795 to 4.803	4.097	4.095 to 4.1	14.137	14.089 to 14.184	7.15	7.147 to 7.153
80–84	5.623	5.617 to 5.628	4.225	4.223 to 4.228	21.392	21.305 to 21.479	8.726	8.722 to 8.73
85–89	6.424	6.416 to 6.431	3.897	3.894 to 3.9	28.545	28.4 to 28.692	9.536	9.53 to 9.541
90–94	6.833	6.822 to 6.844	3.593	3.589 to 3.597	33.043	32.833 to 33.254	9.957	9.95 to 9.965
95 plus	6.830	6.813 to 6.848	3.405	3.399 to 3.411	34.058	33.789 to 34.329	10.015	10.004 to 10.026
Period								
1992–1996	0.805	0.804 to 0.805	0.789	0.789 to 0.789	0.73	0.727 to 0.732	0.809	0.808 to 0.809
1997–2001	0.873	0.873 to 0.874	0.868	0.867 to 0.868	0.84	0.838 to 0.842	0.892	0.891 to 0.892
2002–2006	0.951	0.950 to 0.951	0.945	0.945 to 0.946	0.973	0.972 to 0.975	0.992	0.991 to 0.992
2007–2011	1.027	1.026 to 1.027	1.036	1.036 to 1.036	1.05	1.048 to 1.051	1.032	1.032 to 1.032
2012–2016	1.121	1.120 to 1.121	1.154	1.154 to 1.154	1.171	1.168 to 1.174	1.104	1.104 to 1.105
2017–2021	1.301	1.300 to 1.302	1.292	1.292 to 1.292	1.365		1.228	1.227 to 1.228
Cohort								
1897–1901	4.819	4.782 to 4.856	3.371	3.35 to 3.392	13.26	13.099 to 13.423	7.079	7.059 to 7.099
1902–1906	4.146	4.131 to 4.161	3.089	3.081 to 3.098	10.254	10.164 to 10.344	5.699	5.69 to 5.707
1907–1911	3.525	3.516 to 3.533	2.838	2.834 to 2.843	8.089	8.033 to 8.146	4.711	4.707 to 4.716
1912–1916	2.974	2.969 to 2.980	2.607	2.604 to 2.61	6.58	6.544 to 6.617	4.011	4.008 to 4.014
1917–1921	2.506	2.502 to 2.510	2.392	2.39 to 2.395	5.14	5.118 to 5.162	3.277	3.275 to 3.279
1922–1926	2.205	2.202 to 2.207	2.192	2.19 to 2.194	4.126	4.113 to 4.14	2.767	2.765 to 2.768
1927–1931	1.981	1.978 to 1.983	1.982	1.98 to 1.983	3.467	3.458 to 3.477	2.439	2.438 to 2.44
1932–1936	1.762	1.760 to 1.764	1.789	1.788 to 1.79	2.797	2.789 to 2.805	2.065	2.064 to 2.066
1937–1941	1.575	1.573 to 1.577	1.609	1.608 to 1.61	2.242	2.233 to 2.25	1.744	1.744 to 1.745
1942–1946	1.369	1.367 to 1.370	1.427	1.426 to 1.428	1.739	1.731 to 1.748	1.426	1.425 to 1.427
1947–1951	1.192	1.190 to 1.193	1.268	1.267 to 1.269	1.375	1.367 to 1.383	1.198	1.197 to 1.199
1952–1956	1.065	1.064 to 1.067	1.147	1.146 to 1.148	1.16	1.151 to 1.168	1.066	1.066 to 1.067
1957–1961	0.941	0.940 to 0.943	1.034	1.034 to 1.035	0.943	0.935 to 0.952	0.919	0.918 to 0.919
1962–1966	0.836	0.835 to 0.838	0.925	0.924 to 0.926	0.765	0.757 to 0.773	0.796	0.795 to 0.797
1967–1971	0.749	0.748 to 0.751	0.824	0.824 to 0.825	0.633	0.626 to 0.641	0.699	0.698 to 0.7
1972–1976	0.678	0.677 to 0.680	0.742	0.742 to 0.743	0.52	0.512 to 0.527	0.614	0.613 to 0.615
1977–1981	0.618	0.616 to 0.619	0.669	0.669 to 0.67	0.442	0.435 to 0.45	0.551	0.55 to 0.552
1982–1986	0.561	0.559 to 0.562	0.599	0.598 to 0.599	0.375	0.368 to 0.382	0.493	0.492 to 0.494
1987–1991	0.503	0.501 to 0.504	0.532	0.532 to 0.533	0.315	0.308 to 0.322	0.44	0.44 to 0.441
1992–1996	0.452	0.451 to 0.453	0.47	0.469 to 0.47	0.266	0.26 to 0.271	0.396	0.395 to 0.397
1997–2001	0.404	0.403 to 0.405	0.413	0.413 to 0.414	0.209	0.204 to 0.214	0.334	0.333 to 0.334
2002–2006	0.359	0.357 to 0.360	0.366	0.366 to 0.367	0.163	0.159 to 0.168	0.281	0.28 to 0.281
2007–2011	0.313	0.312 to 0.314	0.324	0.323 to 0.325	0.131	0.127 to 0.135	0.238	0.237 to 0.239
2012–2016	0.270	0.269 to 0.271	0.285	0.284 to 0.286	0.103	0.1 to 0.107	0.196	0.195 to 0.196
2017–2021	0.235	0.233 to 0.237	0.254	0.253 to 0.256	0.071	0.068 to 0.074	0.14	0.139 to 0.14

### 3.4 Relationship between IS burden and SDI levels

**Figure 3:**
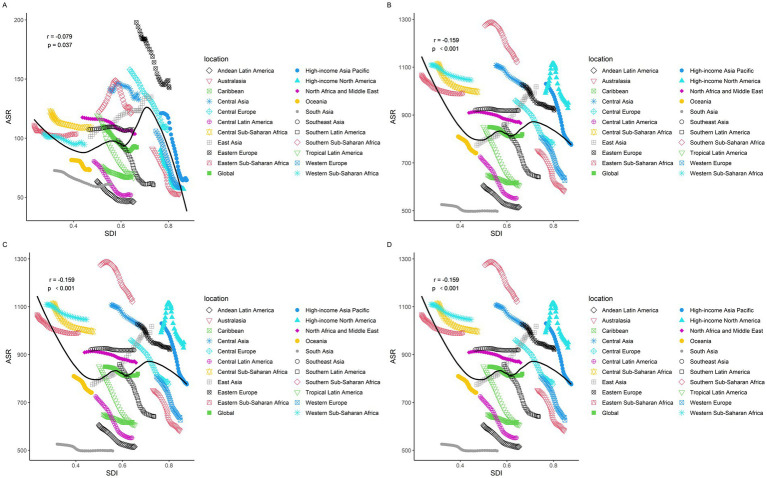
Changes in the ASIR, ASPR, ASMR, and DALYs of gout globally and across different SDI regions. **(A)** ASIR, **(B)** ASPR, **(C)** ASMR, and **(D)** DALYs.

Figure 3 shows that the ASIR (*r* = −0.079, *p* = 0.037), ASPR (*r* = −0.159, *p* < 0.001), ASMR (*r* = −0.103, *p* = 0.006), and ASDR (*r* = −0.151, *p* < 0.001) exhibit a gradual decrease with increasing SDI values. Based on the categorization criteria for SDI in the 2021 GBD, 204 countries and territories worldwide are divided into five levels: high SDI, high-middle SDI, middle SDI, low-middle SDI, and low SDI. To elucidate the relationship between SDI and epidemiological trends in IS more specifically, we described the trends in ASIR, ASPR, ASMR, and DALYs for IS from 1990 to 2021 in countries with different SDI levels. Both ASIR and ASPR show an upward trend in the middle SDI, while individuals in other SDI levels experience a downward trend. The age-standardized mortality and DALYs in all SDI regions decreased from 1990 to 2021 (Table 4).

**Table 4:** The change in ASR of prevalence, incidence, deaths, and DALYs from 1990 to 2021 among different SDI levels.

Region	Incidence		Prevalence		Death		DALYs	
	ASR	EAPC	ASR	EAPC	ASR	EAPC	ASR	EAPC
High SDI	−37.41%	−1.75	−12.84%	−0.57	−63.94%	−3.58	−56.84%	−2.98
High-middle SDI	−17.92%	−0.74	−1.43%	−0.09	−46.68%	−2.4	−42.91%	−2.19
Low SDI	−12.94%	−0.51	−9.82%	−0.39	−13.48%	−0.48	−14.94%	−0.58
Low-middle SDI	−7.65%	−0.34	−1.69%	−0.09	−13.70%	−0.49	−13.94%	−0.52
Middle SDI	6.16%	0.12	9.47%	0.27	−22.42%	−0.86	−21.37%	−0.83

### 3.5 Population attributable fraction of the IS

**Figure 4:**
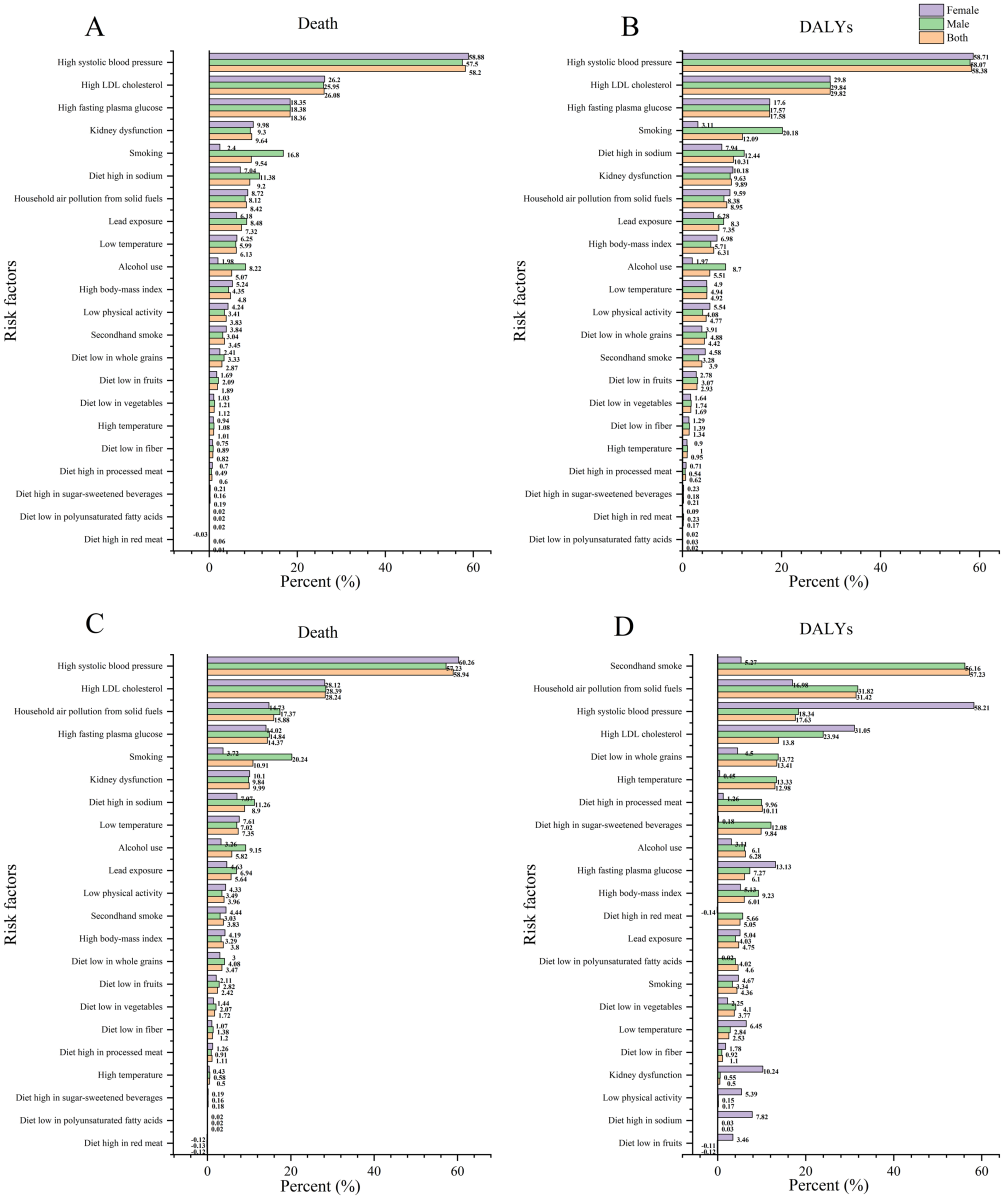
PAF of the IS in 2021 **(A,B)** and 1990 **(C,D)**.

The risk factors for IS-related ASMR and DALYs are divided into three categories: environmental risks, behavioral risks, and metabolic risks. In both 1990 and 2021, the leading risk factor for IS-related ASMR was elevated systolic blood pressure. In 2021, elevated systolic blood pressure was also identified as the primary factor for DALYs in both men and women, with respective percentages of 58.38 and 58.71%. Secondhand smoking was the primary contributor to DALYs, with a PAF of 57.23% (95% CI 43.56–68.28), particularly among males, who exhibited a PAF of 56.16% (95% CI 42.37–67.64). For women, the most significant risk factor for DALYs was identified as elevated systolic blood pressure, with a PAF of 58.21% (95% CI: 44.33 to 69.23) (Figure 4; Table 5; Supplementary Table S4).

**Table 5:** Attributable ASMR and DALY percentages by IS risk factors in 2021 and by sex globally.

	Age-standardized deaths percent (95% UI)			Age-standardized DALYs percent (95% UI)		
	Overall	Male	Female	Overall	Male	Female
All risk	87.6 (81.05 to 92.66)	88.73 (82.83 to 93.36)	86.49 (79.24 to 91.9)	88.51 (82.22 to 92.93)	89.63 (83.85 to 93.77)	87.26 (80.1 to 92.26)
Level 1 risks						
Environmental risks	35.42 (27.69 to 42.8)	37.06 (28.6 to 45.16)	33.79 (27.01 to 40.52)	35.18 (27.44 to 42.51)	36.25 (27.95 to 44.31)	34 (27.06 to 40.71)
Behavioral risks	31.22 (19.37 to 42.69)	40.06 (28.3 to 51.78)	22.52 (10.1 to 34.58)	37.01 (25.17 to 47.66)	45.48 (33.55 to 56.17)	27.59 (16.01 to 39.48)
Metabolic risks	77.17 (66.16 to 85.59)	76.6 (66.08 to 85.39)	77.72 (66.96 to 86.22)	78.19 (67.27 to 86.41)	77.9 (66.83 to 86.16)	78.51 (67.43 to 86.85)
Environmental risks						
Household air pollution from solid fuels	8.42 (4.63 to 16)	8.12 (4.26 to 15.97)	8.72 (4.79 to 16.03)	8.95 (4.98 to 16.7)	8.38 (4.46 to 16.22)	9.59 (5.35 to 17.24)
High temperature	1.01 (0.06 to 2.42)	1.08 (0.08 to 2.59)	0.94 (0.05 to 2.25)	0.95 (0.11 to 2.24)	1 (0.11 to 2.33)	0.9 (0.09 to 2.11)
Low temperature	6.13 (5.34 to 6.9)	5.99 (5.26 to 6.83)	6.25 (5.41 to 7.02)	4.92 (4.25 to 5.69)	4.94 (4.28 to 5.77)	4.9 (4.2 to 5.64)
Lead exposure	7.32 (−0.98 to 16.23)	8.48 (−1.15 to 18.84)	6.18 (−0.81 to 13.67)	7.35 (−0.98 to 16.41)	8.3 (−1.12 to 18.51)	6.28 (−0.82 to 13.98)
Behavioral risks						
Smoking	9.54 (7.76 to 11.63)	16.8 (14 to 19.8)	2.4 (1.84 to 3.04)	12.09 (10.22 to 14.2)	20.18 (17.41 to 23.14)	3.11 (2.53 to 3.76)
Secondhand smoke	3.45 (2.28 to 4.66)	3.04 (2.04 to 4.07)	3.84 (2.57 to 5.22)	3.9 (2.65 to 5.19)	3.28 (2.23 to 4.35)	4.58 (3.08 to 6.11)
Alcohol use	5.07 (−0.68 to 12.58)	8.22 (−1.16 to 20.18)	1.98 (−0.19 to 5.16)	5.51 (−0.84 to 13.52)	8.7 (−1.41 to 21.19)	1.97 (−0.23 to 5.16)
Low physical activity	3.83 (−0.89 to 8.71)	3.41 (−0.17 to 7.5)	4.24 (−1.6 to 10.22)	4.77 (1.3 to 8.92)	4.08 (1.31 to 7.35)	5.54 (1.34 to 10.42)
Metabolic risks						
High systolic blood pressure	58.2 (43.98 to 69.26)	57.5 (42.93 to 69)	58.88 (44.99 to 69.99)	58.38 (44.19 to 69.47)	58.07 (43.36 to 69.48)	58.71 (43.97 to 69.95)
High LDL cholesterol	26.08 (8.1 to 44.61)	25.95 (8.22 to 44.1)	26.2 (8 to 45.16)	29.82 (10.28 to 48.2)	29.84 (10.51 to 47.91)	29.8 (10.17 to 48.55)
High fasting plasma glucose	18.36 (14.53 to 22.54)	18.38 (14.49 to 22.52)	18.35 (14.51 to 22.52)	17.58 (13.9 to 21.58)	17.57 (13.86 to 21.52)	17.6 (13.92 to 21.63)
High body mass index	4.8 (0.7 to 9.32)	4.35 (0.63 to 8.51)	5.24 (0.78 to 10.18)	6.31 (0.95 to 11.95)	5.71 (0.85 to 10.89)	6.98 (1.07 to 13.22)
Kidney dysfunction	9.64 (6.46 to 12.89)	9.3 (6.34 to 12.28)	9.98 (6.45 to 13.52)	9.89 (7.02 to 12.85)	9.63 (6.99 to 12.42)	10.18 (7.14 to 13.26)
A diet high in sodium	9.2 (1.91 to 20.93)	11.38 (2.86 to 23.92)	7.04 (0.92 to 17.29)	10.31 (2.59 to 22.38)	12.44 (3.6 to 25.53)	7.94 (1.43 to 18.77)
A diet high in red meat	0.01 (−0.36 to 0.84)	0.06 (−0.35 to 1.03)	−0.03 (−0.44 to 0.61)	0.17 (−0.19 to 1.64)	0.23 (−0.24 to 1.8)	0.09 (−0.32 to 1.35)
A diet low in whole grains	2.87 (−2.74 to 9.18)	3.33 (−3.25 to 10.19)	2.41 (−2.24 to 8.3)	4.42 (−4.55 to 12.22)	4.88 (−5.09 to 13.22)	3.91 (−3.94 to 11.15)
A diet low in fruits	1.89 (0.5 to 3.49)	2.09 (0.73 to 3.71)	1.69 (0.2 to 3.28)	2.93 (1.59 to 4.6)	3.07 (1.69 to 4.71)	2.78 (1.46 to 4.5)
A diet low in fiber	0.82 (−0.06 to 2.05)	0.89 (−0.05 to 2.09)	0.75 (−0.15 to 2.04)	1.34 (−0.06 to 2.77)	1.39 (−0.06 to 2.77)	1.29 (−0.05 to 2.79)
A diet low in vegetables	1.12 (0.21 to 1.99)	1.21 (0.39 to 2.09)	1.03 (0.03 to 1.97)	1.69 (0.89 to 2.54)	1.74 (1 to 2.54)	1.64 (0.8 to 2.56)
A diet low in polyunsaturated fatty acids	0.02 (0.01 to 0.04)	0.02 (0.01 to 0.04)	0.02 (0.01 to 0.04)	0.02 (0.01 to 0.05)	0.03 (0.01 to 0.05)	0.02 (0.01 to 0.04)
A diet high in sugar-sweetened beverages	0.19 (0.1 to 0.29)	0.16 (0.08 to 0.25)	0.21 (0.11 to 0.33)	0.21 (0.1 to 0.32)	0.18 (0.09 to 0.28)	0.23 (0.12 to 0.36)
A diet high in processed meat	0.6 (0.15 to 1.04)	0.49 (0.12 to 0.85)	0.7 (0.17 to 1.24)	0.62 (0.16 to 1.06)	0.54 (0.14 to 0.93)	0.71 (0.18 to 1.23)

### 3.6 Decomposition analysis of IS

**Figure 5:**
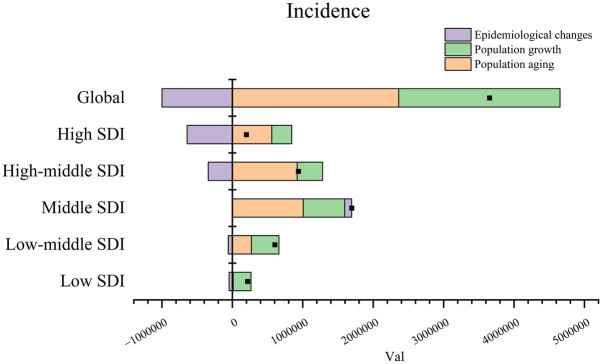
Decomposition analysis of IS incidence from 1990 to 2021.

Decomposition analyses provide insights into the influence of population aging, population growth, and epidemiology on the burden of ischemic stroke (IS). From 1990 to 2021, there was a notable increase in the incidence of IS globally and across the five SDI regions. This increase can be primarily attributed to population growth at the demographic level. Over the past three decades, population growth has accounted for 64.7, 98.11, and 59.31% of the observed increase in incidence rates globally, in the medium-high SDI region, and in the medium SDI region, respectively (Table 6). The impact of population growth on morbidity was most significant in the high SDI region (142.82%), followed by the low SDI region (116.49%), and then the low and medium SDI regions (65.3%) (Figure 5; Table 6).

**Table 6:** Analysis of IS incidence decomposition from 1990 to 2021.

Location	Overall_difference	Population aging_effect	Population growth_effect	Epidemiological changes_effect	Population aging (%)	Population growth (%)	Epidemiological changes (%)	1990 val	2019 val	Difference
Global	3652471.33	2363100.974	2289868.863	−1000498.511	64.7%	62.69%	−27.39%	4,151,978	7,804,449	3,652,471
High SDI	198538.31	559127.463	283549.632	−644138.786	281.62%	142.82%	−324.44%	1153374.2	1351912.5	198538.3
High-middle SDI	937063.34	919335.652	361260.816	−343533.13	98.11%	38.55%	−36.66%	1306276.5	2243339.9	937063.3
Middle SDI	1694984.06	1005375.67	588911.494	100696.901	59.31%	34.74%	5.94%	953540.6	2648524.6	1694984.1
Low-middle SDI	603576.33	269433.782	392512.929	−58370.385	44.64%	65.03%	−9.67%	516850.9	1120427.2	603576.3
Low SDI	216804.92	11360.959	252560.325	−47116.36	5.24%	116.49%	−21.73%	216493.5	433298.4	216804.9

### 3.7 Frontier analysis of IS DALYs

**Figure 6:**
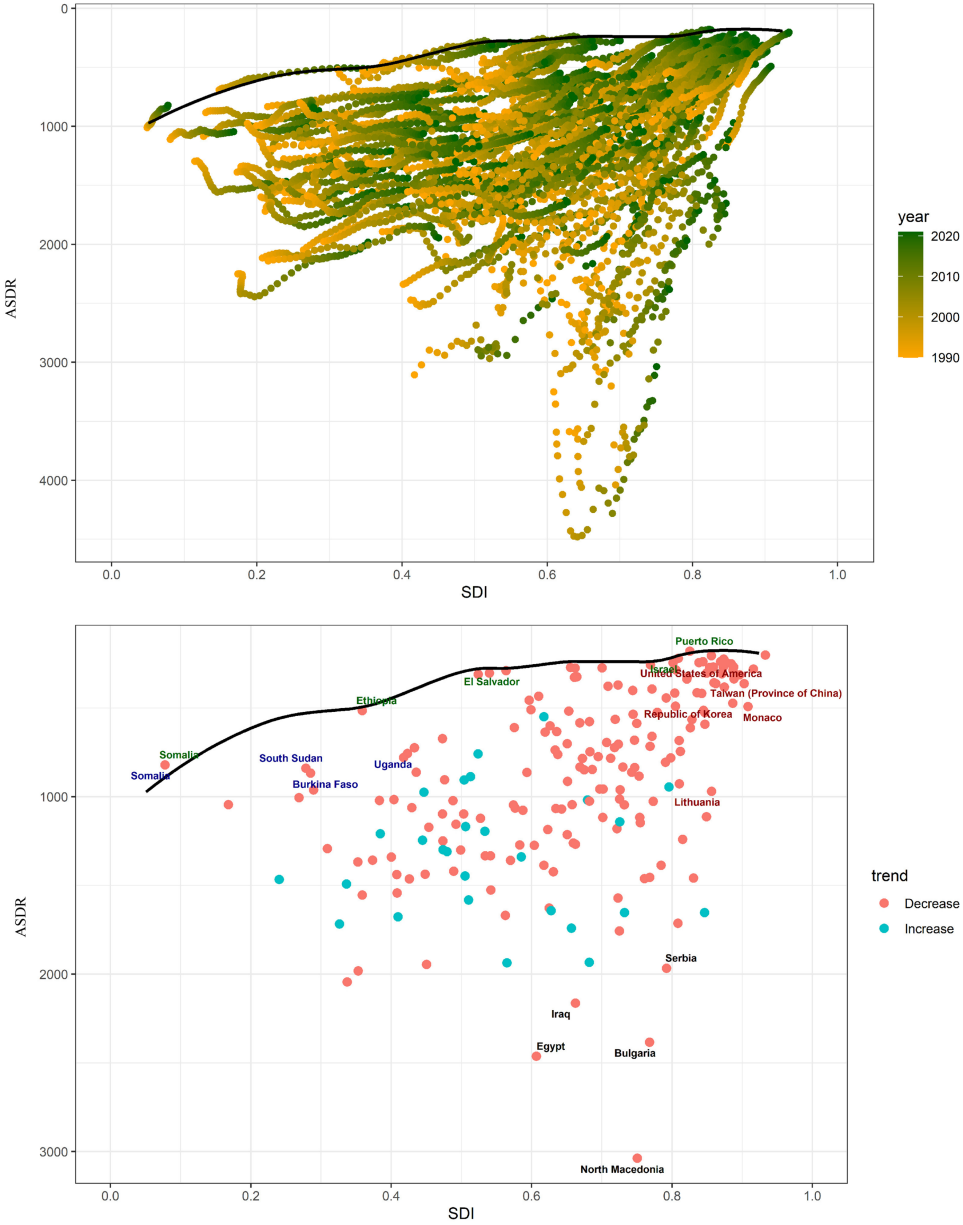
Frontier analysis using ASDR. The solid black line indicates the frontier, while the dots indicate the countries and regions. The red dots show a downward trend in IS ASDR from 1990 to 2021, whereas the blue dots indicate the opposite trend.

The effective difference (EF) and variance were observed to be relatively minor in the low SDI region, while the EF and variance exhibited notable discrepancies in the high SDI region. Additionally, our observations indicated a notable degree of heterogeneity in the EF when the SDI was between 0.6 and 0.8. The five countries closest to the frontier were Somalia, Puerto Rico, Ethiopia, Israel, and El Salvador. In contrast, the five countries furthest from the frontier were North Macedonia, Egypt, Bulgaria, Iraq, and Serbia (see Figure 6).

### 3.8 Prediction analysis

**Figure 7:**
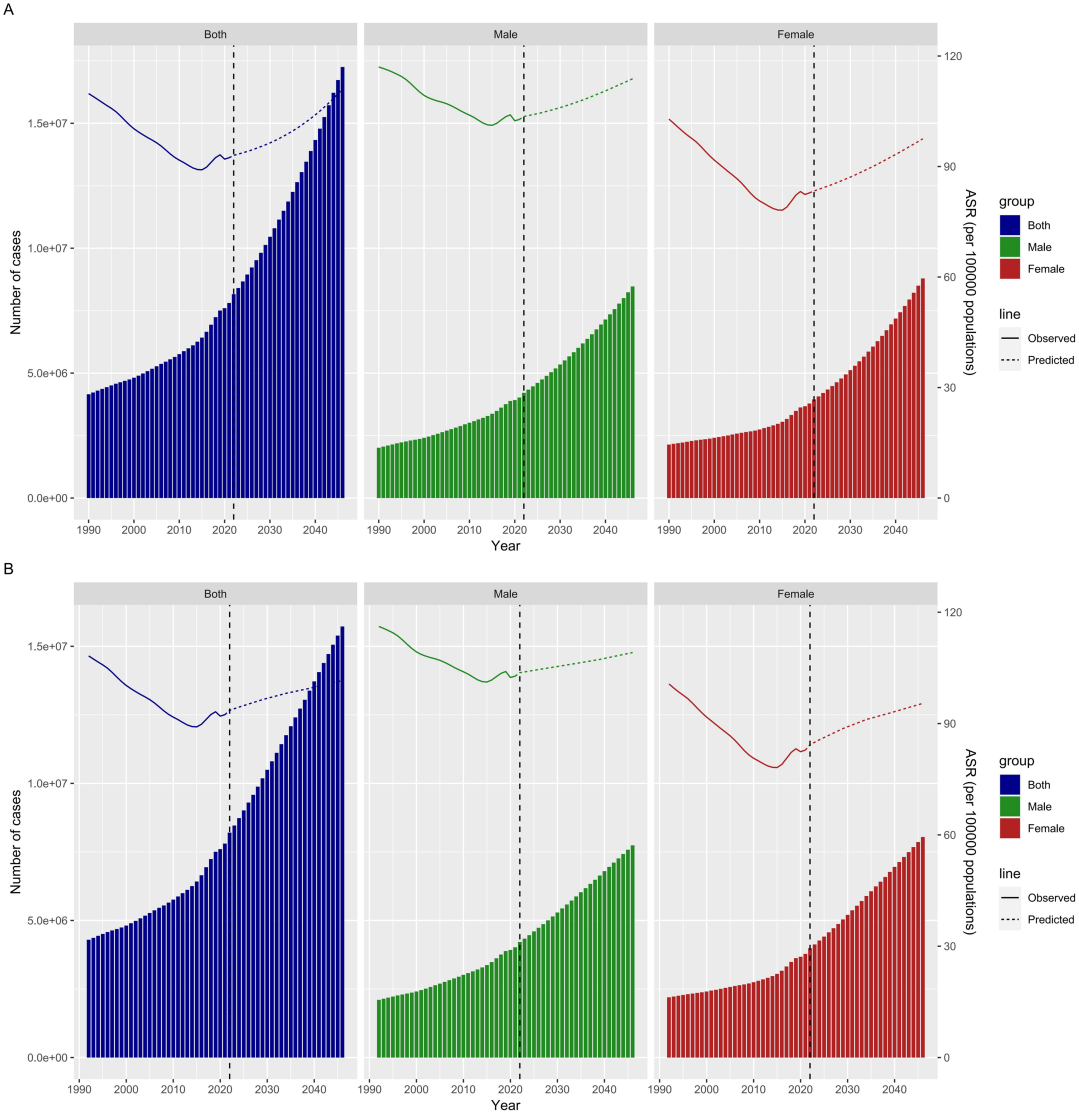
Projected ASIR for IS by gender (both men and women) from 1990 to 2046 based on the BAPC model **(A)** and Norpred **(B)**.

To delineate and forecast the incidence trends of IS up to 2046, the BAPC model and Norpred were employed. A notable increase in the number of incidences was observed for both genders from 1990 to 2046, as indicated by the BAPC model. However, the ASIR demonstrated a fluctuating trend, initially declining and then increasing, with females showing a more pronounced trajectory. Specifically, the ASIR declined from 1990 to 2015. This downward trend reversed between 2016 and 2021, with projections indicating its continuation until 2046 (Figure 7A). The trend predicted by Norpred was also consistent with that of the BAPC (Figure 7B).

## 4 Discussion

Our research provided the most up-to-date description and analysis of global epidemiological trends in IS incidence, prevalence, mortality, and DALYs based on the latest data from the 2021 GBD. From 1990 to 2021, the incidence, prevalence, mortality, and DALYs for IS showed a consistent upward trajectory. However, a downward trend was observed in the ASIR, ASPR, ASMR, and DALYs. This finding aligns with the observations made by Zhang et al. in their research using the GBD 2019. The decline in mortality and DALYs for IS may be attributed to several factors. First, intensive care management methods for patients with severe strokes are becoming increasingly sophisticated (18). Second, the use of tissue fibrinogen activators in treatment regimens is also a significant contributing factor (19). Additionally, advances in endovascular thrombectomy may also play a role. In conclusion, these factors suggest that the observed trends result from a complex interplay of systemic improvements in stroke management (12). Moreover, the epidemiological trend of IS in 2021 showed variations related to age and gender. In particular, age-specific rates of incidence, prevalence, mortality, and DALYs for IS demonstrated an overall increase with age, a finding corroborated by previous research. This trend has also been confirmed by previous studies (20). The aging population appears to significantly contribute to the rising incidence. A total of 85% of strokes occur in individuals aged 65 and older, and the incidence of stroke doubles every decade after the age of 55 (21). The molecular mechanisms of the immune system dysregulate with age, affecting the pathophysiology and transcriptional amplitude in stroke patients, thereby playing a crucial role in determining the incidence and outcomes of stroke (22).

As we age, there is an imbalance between pro-inflammatory and anti-inflammatory molecules, with the former becoming more predominant. This low-grade, chronic inflammatory state heightens the risk of stroke. Additionally, the aging immune system may provoke potentially harmful immune responses following a stroke, accelerate neurodegeneration, and significantly influence stroke prognosis (23, 24). Elderly patients who have experienced a cerebrovascular accident are more susceptible to immune deficiencies, vascular dysfunction, and a higher risk of infections (25). Furthermore, the aging process is linked to larger cerebral infarcts, worsening neurological deficits post-stroke, and damage to the blood–brain barrier (26). These factors can explain the increase in ASIR, ASPR, ASMR, and DALYs of IS in 2021 with age.

The SDI is often used to gauge economic advancement within a region or country. Studies have shown that the epidemiological patterns of IS may change according to economic development levels (27). Therefore, we conducted a comprehensive analysis of the relationship between the epidemiological trends of IS and SDI levels. We observed that SDI levels were negatively associated with ASIR, ASPR, ASMR, and DALYs in IS. In general, SDI, ASIR, and ASPR of IS exhibited an upward trend, while ASMR and DALYs showed a downward trend. Authoritative statistics show that low- and middle-income countries have higher stroke mortality rates and tend to experience lower ages of onset, accounting for a relatively high proportion of strokes (19–30%), exacerbating the burden of disability and DALYs lost in developing countries (4, 28, 29).

Identifying risk factors is crucial in preventing IS. The primary risk factor for IS-related death and the global DALY rate in 2021 was high systolic blood pressure. Systolic blood pressure (SBP) is linked to various cardiovascular outcomes, including ischemic heart disease, with high SBP being the most significant contributor to SBP-related deaths (30–32). It has been demonstrated that DALYs and deaths associated with elevated SBP were increased. At the global level, 26% of DALYs occurred in individuals with SBP between 140 and 150 mmHg, while 45% occurred in those with SBP of 160 mmHg or higher. Individuals with high SBP (140 mmHg or higher) account for 73.2% (95% UI, 71. 5%-75. 0%) of all SBP-related deaths (33). Consequently, managing blood pressure is critical for IS prevention. Strategies include implementing community-level blood pressure screening programs for high-risk groups; developing mobile health applications to help patients monitor and manage their blood pressure; reducing salt in processed foods; and conducting public education campaigns. Additionally, we identified high LDL cholesterol and elevated fasting blood glucose as significant risk factors. To address these, we should promote cholesterol screening, conduct educational programs to guide high-risk individuals in managing lipid levels, implement early diabetes screening and prevention plans, and offer comprehensive management for diabetes patients, including diet, exercise, and medication. Environmental factors also play a critical role, with household air pollution from solid fuels being a major contributor. To mitigate this, we can promote clean cooking technologies and fuels, especially in low-income and rural areas, phase out high-pollution fuels, and conduct public education campaigns to raise awareness about the dangers of indoor air pollution.

Frontier analysis, based on the SDI and age-standardized DALYs, provided key insights into the trajectory of global IS DALYs from 1990 to 2021. The overall narrative presents a clear picture: the burden of DALYs due to IS increased as the SDI rose. However, an in-depth examination of the 2021 data revealed subtle differences across the various SDI regions. In the low-SDI region, Somalia, Ethiopia, and El Salvador showed limited potential for DALY improvement. Among all the regions, North Macedonia has the greatest potential for enhancing DALYs, and local governments should implement additional relevant measures to foster DALY improvements.

In the prediction analysis, the age-standardized rate and numbers of new cases for IS from 2020 to 2046 showed a gradual increasing trend. Population aging may provide a more plausible explanation for the phenomenon. The 20th century witnessed an unprecedented increase in average life expectancy and a rapid decline in human fertility in many countries worldwide. Globally, the proportion of the population older than 60 years increased from 9.2% in 1990 to 11.7% in 2013 and is projected to reach 21.1% by 2050 (34). Among the 293 GBD causes, IS was identified as an age-related diseases (35). 85% of strokes occur in individuals over 65 years of age, and the incidence of stroke doubles every decade after the age of 55 (21). Therefore, the rates of aging are accelerating, leading to an increased burden of IS. The burden of age-related diseases accounts for 51.3% of the overall burden (35). Based on the World Health Organization’s concept of healthy aging, each country should develop a monitoring and evaluation framework tailored to its own national realities, providing vital references for optimizing prevention and control strategies.

### 4.1 Limitations

This study’s reliance on the GBD database presents several limitations. As a secondary data source, our analysis may reflect the biases inherent in GBD’s data collection and modeling techniques. Although GBD provides global health metrics, its applicability is limited by variations in national health systems and data collection capabilities. Additionally, GBD might not fully capture local health trends, particularly in areas with limited data availability. Delays in data updates can also impact the timeliness of results, especially in a rapidly evolving global health context. Therefore, it is important to interpret and apply the findings with caution and, whenever possible, validate them using other data sources.

## Data Availability

The datasets presented in this study can be found in online repositories. The names of the repository/repositories and accession number(s) can be found in the article/Supplementary material.

## Footnotes

^1^ https://www.healthdata.org/research-analysis/gbd
