# Supplementary material for: Comprehensive analysis of the ischemic stroke burden at global, regional, and national levels (1990–2021): trends, influencing factors, and future projections
Source: Front Neurol. 2025 Mar 19;16:1492691. doi: 10.3389/fneur.2025.1492691 (PMC11961430; doi:10.3389/fneur.2025.1492691)
Supplement: Supplementary file 1 [file Table_1.DOCX]

TS 1. Numbers and ASR per 100 000 cases of prevalence of ischemic stroke in 1990 and 2021, along with the relative changes and EAPC in ASR per 100 000 cases from 1990 to 2021, categorized by global, SDI, and GBD regions.

| **Characteristic** | **Number in 1990 (95% CI)** | **Age-standardized rate in 1990 (95% CI)** | **Number in 2021 (95% CI)** | **Age-standardized rate in 2021 (95% CI)** | **Relative change of numbers from 1990 to 2021** | **Relative change of age-standardized rate from 1990 to 2021** | **EAPC (Age-standardized rate, 95% CI)** |
| --- | --- | --- | --- | --- | --- | --- | --- |
| Andean Latin America | 138243 (131027 to 145937) | 604.93 (573.4 to 638.13) | 312485 (297506 to 327200) | 515.11 (490.59 to 538.97) | 126.04% | -14.85% | -0.6 (-0.64 to -0.57) |
| Australasia | 176175 (169750 to 183601) | 751.35 (724.29 to 783.41) | 296951 (286363 to 308252) | 581.91 (560.18 to 603.18) | 68.55% | -22.55% | -0.93 (-0.98 to -0.89) |
| Caribbean | 178810 (169860 to 188129) | 649.08 (616.1 to 684.12) | 327165 (312541 to 341548) | 616.95 (589.7 to 643.62) | 82.97% | -4.95% | -0.18 (-0.2 to -0.16) |
| Central Asia | 550460 (523473 to 577217) | 1107.87 (1052.26 to 1164.94) | 866712 (829519 to 902763) | 1014.86 (969.9 to 1063.57) | 57.45% | -8.40% | -0.31 (-0.33 to -0.29) |
| Central Europe | 1418232 (1324818 to 1513620) | 959.62 (897.79 to 1022.43) | 1631106 (1537835 to 1733827) | 776.07 (733.54 to 820.85) | 15.01% | -19.13% | -0.77 (-0.82 to -0.72) |
| Central Latin America | 678692 (630062 to 725121) | 725.24 (673.94 to 777.83) | 1387552 (1296756 to 1484316) | 552.56 (516.91 to 591.3) | 104.45% | -23.81% | -1.03 (-1.09 to -0.96) |
| Central Sub-Saharan Africa | 266924 (253028 to 281576) | 1113.57 (1052.23 to 1178.44) | 596223 (568898 to 624229) | 995.72 (947.71 to 1046.32) | 123.37% | -10.58% | -0.41 (-0.43 to -0.38) |
| East Asia | 6921708 (6208387 to 7617524) | 774.23 (691.49 to 863.72) | 21503823 (19292836 to 23714701) | 1017.96 (919.97 to 1120.07) | 210.67% | 31.48% | 0.95 (0.89 to 1) |
| Eastern Europe | 2869099 (2565925 to 3174157) | 1028.01 (921.85 to 1132.82) | 3049360 (2763322 to 3340261) | 920.45 (835.8 to 1005.34) | 6.28% | -10.46% | -0.38 (-0.41 to -0.35) |
| Eastern Sub-Saharan Africa | 867801 (811694 to 925063) | 1066.61 (996.15 to 1139.76) | 1869299 (1761941 to 1973402) | 992.11 (934.22 to 1049.93) | 115.41% | -6.99% | -0.27 (-0.29 to -0.25) |
| Global | 34668041 (32153637 to 37171588) | 849.49 (785.92 to 913.25) | 69944885 (64788695 to 75009603) | 819.47 (760.26 to 878.71) | 101.76% | -3.53% | -0.18 (-0.21 to -0.16) |
| High SDI | 10141956 (9487690 to 10833008) | 933.82 (874.48 to 998.01) | 15864865 (14925798 to 16872855) | 813.89 (768.62 to 864.1) | 56.43% | -12.84% | -0.57 (-0.64 to -0.5) |
| High-income Asia Pacific | 2058456 (1893145 to 2220222) | 1030.51 (947.08 to 1109.23) | 3295962 (3048493 to 3555086) | 775.63 (722.99 to 832.15) | 60.12% | -24.73% | -1.04 (-1.09 to -0.98) |
| High-income North America | 3403855 (3107282 to 3724886) | 991.52 (904.49 to 1081.88) | 5699098 (5260863 to 6152298) | 949.99 (880.58 to 1024.5) | 67.43% | -4.19% | -0.39 (-0.57 to -0.21) |
| High-middle SDI | 9174385 (8429846 to 9882568) | 906.21 (834.66 to 975.02) | 17110640 (15738888 to 18478803) | 893.25 (824.01 to 959.98) | 86.50% | -1.43% | -0.09 (-0.14 to -0.04) |
| Low SDI | 2124876 (1976570 to 2270871) | 838.06 (776.26 to 901.21) | 4447699 (4174215 to 4699709) | 755.73 (707.71 to 800.06) | 109.32% | -9.82% | -0.39 (-0.43 to -0.36) |
| Low-middle SDI | 4631395 (4229094 to 5023597) | 680.79 (620.57 to 741.66) | 10234283 (9418964 to 11004022) | 669.3 (616.25 to 720.63) | 120.98% | -1.69% | -0.09 (-0.11 to -0.06) |
| Middle SDI | 8555790 (7763207 to 9309792) | 765.88 (693.01 to 837.51) | 22230671 (20239639 to 24119856) | 838.38 (764.78 to 911.01) | 159.83% | 9.47% | 0.27 (0.24 to 0.3) |
| North Africa and Middle East | 1847829 (1741882 to 1963338) | 909.31 (852.4 to 967.58) | 4366507 (4157818 to 4571158) | 866.3 (823.67 to 909.4) | 136.30% | -4.73% | -0.18 (-0.2 to -0.17) |
| Oceania | 26485 (25138 to 27927) | 809.68 (767.68 to 854.97) | 59506 (57089 to 62156) | 741.44 (711.42 to 771.98) | 124.68% | -8.43% | -0.31 (-0.31 to -0.3) |
| South Asia | 3441863 (3041705 to 3832963) | 526.01 (462.25 to 586.94) | 7828397 (6997065 to 8621267) | 497.74 (445.71 to 549.03) | 127.45% | -5.37% | -0.21 (-0.25 to -0.18) |
| Southeast Asia | 2557636 (2350464 to 2762167) | 921.18 (846.81 to 999.59) | 6104966 (5651319 to 6562857) | 919.03 (851.92 to 986.33) | 138.70% | -0.23% | -0.03 (-0.04 to -0.02) |
| Southern Latin America | 399118 (380518 to 419432) | 859.38 (819.62 to 903.12) | 546569 (524189 to 570536) | 640.57 (614.79 to 668.14) | 36.94% | -25.46% | -1.05 (-1.1 to -1) |
| Southern Sub-Saharan Africa | 364210 (330935 to 399540) | 1274.58 (1149.82 to 1404.06) | 640170 (583548 to 694855) | 1121.97 (1019.94 to 1224.88) | 75.77% | -11.97% | -0.49 (-0.54 to -0.44) |
| Tropical Latin America | 827664 (739294 to 917729) | 850.01 (757.77 to 943.57) | 1543560 (1392395 to 1692608) | 605.71 (546.63 to 663.92) | 86.50% | -28.74% | -1.24 (-1.3 to -1.18) |
| Western Europe | 4563751 (4343953 to 4819225) | 806.89 (768.41 to 850.59) | 5540066 (5333482 to 5752362) | 625.74 (600.05 to 649.86) | 21.39% | -22.45% | -0.86 (-0.89 to -0.83) |
| Western Sub-Saharan Africa | 1111031 (1034246 to 1188453) | 1109.41 (1026.89 to 1194.52) | 2479408 (2331911 to 2621733) | 1045.98 (977.73 to 1112.64) | 123.16% | -5.72% | -0.23 (-0.24 to -0.21) |

EAPC, Estimated Annual Percentage Change; ASR, Age-standardized rate;
