# Supplementary material for: Comprehensive analysis of the ischemic stroke burden at global, regional, and national levels (1990–2021): trends, influencing factors, and future projections
Source: Front Neurol. 2025 Mar 19;16:1492691. doi: 10.3389/fneur.2025.1492691 (PMC11961430; doi:10.3389/fneur.2025.1492691)
Supplement: Supplementary file 2 [file Table_2.DOCX]

TS 2. Numbers and ASR per 100 000 cases of death of ischemic stroke in 1990 and 2021, along with the relative changes and EAPC in ASR per 100 000 cases from 1990 to 2021, categorized by global, SDI, and GBD regions.

| **Characteristic** | **Number in 1990 (95% CI)** | **Age-standardized rate in 1990 (95% CI)** | **Number in 2021 (95% CI)** | **Age-standardized rate in 2021 (95% CI)** | **Relative change of numbers from 1990 to 2021** | **Relative change of age-standardized rate from 1990 to 2021** | **EAPC (Age-standardized rate, 95% CI)** |
| --- | --- | --- | --- | --- | --- | --- | --- |
| Andean Latin America | 5510 (4930 to 6092) | 31.72 (28.45 to 34.97) | 9794 (8202 to 11614) | 17.73 (14.85 to 21.01) | 77.75% | -44.11% | -2.11 (-2.31 to -1.92) |
| Australasia | 9883 (8880 to 10633) | 45.74 (40.52 to 49.31) | 9400 (7588 to 10375) | 14.11 (11.44 to 15.55) | -4.89% | -69.16% | -4.01 (-4.11 to -3.9) |
| Caribbean | 11986 (11099 to 12742) | 52.96 (48.88 to 56.13) | 19948 (17601 to 22403) | 36.42 (32.14 to 40.94) | 66.42% | -31.24% | -1.16 (-1.24 to -1.09) |
| Central Asia | 32986 (30491 to 35153) | 81.73 (74.95 to 87.16) | 44870 (40632 to 48966) | 70.96 (64.29 to 77.28) | 36.03% | -13.18% | -0.87 (-1.11 to -0.63) |
| Central Europe | 181353 (171927 to 186859) | 140.97 (132.21 to 145.88) | 160200 (144922 to 171306) | 65.59 (59.35 to 70.12) | -11.66% | -53.47% | -2.79 (-2.92 to -2.65) |
| Central Latin America | 24285 (22793 to 25098) | 37.5 (34.9 to 38.88) | 43027 (37956 to 47740) | 18.68 (16.48 to 20.72) | 77.17% | -50.19% | -2.39 (-2.56 to -2.23) |
| Central Sub-Saharan Africa | 8561 (6432 to 10968) | 64.74 (49.96 to 81.82) | 18859 (13940 to 25745) | 59.61 (44.16 to 81.52) | 120.28% | -7.93% | -0.41 (-0.46 to -0.36) |
| East Asia | 442486 (376804 to 522374) | 74.59 (64.1 to 87.38) | 1202218 (1010916 to 1397915) | 63.18 (52.91 to 73.15) | 171.70% | -15.30% | -0.52 (-0.76 to -0.27) |
| Eastern Europe | 405262 (383950 to 415284) | 168.09 (157.84 to 173) | 329291 (299911 to 356035) | 90.99 (82.79 to 98.48) | -18.75% | -45.87% | -2.78 (-3.24 to -2.32) |
| Eastern Sub-Saharan Africa | 24975 (20419 to 31171) | 51.86 (42.85 to 64.28) | 51923 (43067 to 61814) | 46.35 (38.28 to 55.34) | 107.90% | -10.64% | -0.44 (-0.48 to -0.41) |
| Global | 2317112 (2131460 to 2475546) | 73.15 (66.36 to 77.94) | 3591499 (3213281 to 3888327) | 44.18 (39.29 to 47.81) | 55.00% | -39.60% | -1.83 (-1.92 to -1.74) |
| High SDI | 595528 (535847 to 623760) | 53.85 (48.15 to 56.57) | 507950 (426777 to 553062) | 19.42 (16.54 to 21.03) | -14.71% | -63.94% | -3.58 (-3.71 to -3.44) |
| High-income Asia Pacific | 105659 (93891 to 112114) | 62.85 (54.94 to 67.16) | 112785 (87155 to 127258) | 15.77 (12.54 to 17.52) | 6.74% | -74.91% | -4.76 (-4.91 to -4.6) |
| High-income North America | 110127 (96140 to 117560) | 29.57 (25.75 to 31.61) | 126353 (103455 to 138349) | 16.76 (13.85 to 18.27) | 14.73% | -43.33% | -2.38 (-2.68 to -2.08) |
| High-middle SDI | 887525 (831447 to 925960) | 112.05 (103.37 to 116.96) | 1151655 (1025972 to 1263429) | 59.75 (52.99 to 65.45) | 29.76% | -46.68% | -2.4 (-2.6 to -2.2) |
| Low SDI | 87275 (72465 to 110946) | 57.07 (47.48 to 72.01) | 174655 (149333 to 216891) | 49.38 (42.13 to 60.35) | 100.12% | -13.48% | -0.48 (-0.54 to -0.42) |
| Low-middle SDI | 258771 (227017 to 300182) | 58.98 (51.59 to 67.65) | 581649 (517711 to 657998) | 50.9 (45.37 to 57) | 124.77% | -13.70% | -0.49 (-0.53 to -0.44) |
| Middle SDI | 484059 (438723 to 543563) | 66.56 (59.51 to 74.59) | 1171548 (1037070 to 1296280) | 51.64 (45.4 to 57.09) | 142.03% | -22.42% | -0.86 (-0.95 to -0.76) |
| North Africa and Middle East | 131135 (114693 to 148487) | 106.62 (92.27 to 120.78) | 253284 (220812 to 283441) | 73.69 (63.97 to 82.06) | 93.15% | -30.88% | -1.17 (-1.23 to -1.11) |
| Oceania | 806 (627 to 1041) | 48.66 (39.3 to 62.05) | 1843 (1488 to 2389) | 40.7 (33.33 to 52.33) | 128.75% | -16.36% | -0.69 (-0.76 to -0.63) |
| South Asia | 172617 (142120 to 218916) | 43.32 (35.65 to 54.1) | 441296 (382997 to 539467) | 37.98 (33.12 to 45.77) | 155.65% | -12.33% | -0.57 (-0.68 to -0.45) |
| Southeast Asia | 131563 (114808 to 147289) | 72.59 (62.95 to 81.65) | 341541 (293259 to 391953) | 68.09 (58.72 to 77.47) | 159.60% | -6.20% | -0.13 (-0.28 to 0.02) |
| Southern Latin America | 23300 (21541 to 24644) | 57.53 (52.69 to 60.88) | 19658 (17471 to 21165) | 21.14 (18.83 to 22.77) | -15.63% | -63.25% | -2.87 (-3.02 to -2.73) |
| Southern Sub-Saharan Africa | 9795 (8050 to 11101) | 47.29 (38.49 to 53.89) | 24959 (22677 to 27158) | 60.31 (54.56 to 65.54) | 154.81% | 27.54% | 0.94 (0.47 to 1.42) |
| Tropical Latin America | 55341 (50934 to 57541) | 80.36 (72.6 to 84.33) | 71582 (63128 to 76435) | 29.61 (26.03 to 31.67) | 29.35% | -63.15% | -3.02 (-3.14 to -2.91) |
| Western Europe | 380195 (341008 to 399353) | 62.78 (55.96 to 66.13) | 215656 (175955 to 236146) | 16.72 (13.85 to 18.2) | -43.28% | -73.37% | -4.43 (-4.57 to -4.29) |
| Western Sub-Saharan Africa | 49287 (38585 to 64038) | 76.45 (60.17 to 98.52) | 93012 (77668 to 111241) | 68.24 (58.16 to 80.98) | 88.72% | -10.75% | -0.36 (-0.45 to -0.27) |

EAPC, Estimated Annual Percentage Change; ASR, Age-standardized rate;
