# Supplementary material for: Comprehensive analysis of the ischemic stroke burden at global, regional, and national levels (1990–2021): trends, influencing factors, and future projections
Source: Front Neurol. 2025 Mar 19;16:1492691. doi: 10.3389/fneur.2025.1492691 (PMC11961430; doi:10.3389/fneur.2025.1492691)
Supplement: Supplementary file 3 [file Table_3.DOCX]

TS3. Numbers and ASR per 100 000 cases of DALYs of ischemic stroke in 1990 and 2021, along with the relative changes and EAPC in ASR per 100 000 cases from 1990 to 2021, categorized by global, SDI, and GBD regions.

| **Characteristic** | **Number in 1990 (95% CI)** | **Age-standardized rate in 1990 (95% CI)** | **Number in 2021 (95% CI)** | **Age-standardized rate in 2021 (95% CI)** | **Relative change of numbers from 1990 to 2021** | **Relative change of age-standardized rate from 1990 to 2021** | **EAPC (Age-standardized rate, 95% CI)** |
| --- | --- | --- | --- | --- | --- | --- | --- |
| Andean Latin America | 113707 (101758 to 126129) | 574.17 (516.58 to 631.29) | 183296 (154621 to 214987) | 320.11 (270.13 to 375.28) | 61.20% | -44.25% | -2.11 (-2.31 to -1.91) |
| Australasia | 162898 (149974 to 174032) | 714.14 (655.92 to 763.6) | 149125 (129769 to 166039) | 249.45 (216.51 to 278.22) | -8.45% | -65.07% | -3.61 (-3.75 to -3.48) |
| Caribbean | 224864 (209624 to 241939) | 904.27 (839.81 to 968.96) | 353682 (313629 to 399101) | 656.53 (582.42 to 741.98) | 57.29% | -27.40% | -0.97 (-1.04 to -0.9) |
| Central Asia | 719856 (669626 to 766900) | 1625.39 (1511.24 to 1730.11) | 985309 (892229 to 1076484) | 1356.09 (1234.19 to 1474.78) | 36.88% | -16.57% | -1.04 (-1.3 to -0.78) |
| Central Europe | 3203848 (3074577 to 3318844) | 2300.32 (2201.64 to 2386.78) | 2593398 (2385309 to 2776480) | 1101.6 (1014.18 to 1180.5) | -19.05% | -52.11% | -2.7 (-2.83 to -2.57) |
| Central Latin America | 487685 (464998 to 508604) | 642.81 (610.09 to 669.72) | 804658 (727628 to 889436) | 336.61 (304.38 to 372.04) | 65.00% | -47.63% | -2.29 (-2.46 to -2.11) |
| Central Sub-Saharan Africa | 225424 (175337 to 276839) | 1219.92 (970.28 to 1523.56) | 447161 (347102 to 579987) | 1076.29 (830.85 to 1416.65) | 98.36% | -11.77% | -0.54 (-0.59 to -0.49) |
| East Asia | 10279514 (8853619 to 12005870) | 1382.7 (1191.08 to 1606.73) | 24021156 (20420316 to 27562229) | 1165.93 (998.23 to 1336.56) | 133.68% | -15.68% | -0.53 (-0.71 to -0.35) |
| Eastern Europe | 7422390 (7152142 to 7643098) | 2825.98 (2711.24 to 2913.4) | 5713718 (5294961 to 6142848) | 1601.2 (1483.51 to 1723.12) | -23.02% | -43.34% | -2.61 (-3.06 to -2.16) |
| Eastern Sub-Saharan Africa | 616225 (510230 to 758430) | 981.75 (824.06 to 1188.93) | 1231675 (1050099 to 1440686) | 873.5 (749.02 to 1018.61) | 99.87% | -11.03% | -0.47 (-0.51 to -0.44) |
| Global | 46176240 (42961948 to 49414586) | 1286.31 (1195.19 to 1376.06) | 70357912 (64329576 to 76007063) | 837.36 (763.73 to 904.98) | 52.37% | -34.90% | -1.59 (-1.68 to -1.5) |
| High SDI | 10239252 (9461539 to 10819068) | 916.44 (845.06 to 969.25) | 8975176 (7913835 to 9812024) | 395.56 (352.17 to 434.79) | -12.35% | -56.84% | -2.98 (-3.11 to -2.85) |
| High-income Asia Pacific | 1927883 (1766028 to 2055873) | 1047.13 (954.83 to 1117.76) | 1862337 (1582664 to 2083583) | 335.36 (287.81 to 378.86) | -3.40% | -67.97% | -3.99 (-4.14 to -3.83) |
| High-income North America | 2017603 (1818786 to 2187490) | 554.83 (500.04 to 602.52) | 2384648 (2094174 to 2640685) | 352.61 (309.35 to 392.65) | 18.19% | -36.45% | -1.94 (-2.17 to -1.7) |
| High-middle SDI | 16876355 (15868635 to 17770724) | 1885.84 (1768.07 to 1980.92) | 21054343 (19044778 to 23026603) | 1076.54 (973.38 to 1176.25) | 24.76% | -42.91% | -2.19 (-2.39 to -1.99) |
| Low SDI | 2126089 (1791117 to 2645820) | 1074.89 (910.35 to 1345.18) | 4059455 (3492447 to 4963612) | 914.28 (789.76 to 1116.62) | 90.94% | -14.94% | -0.58 (-0.63 to -0.53) |
| Low-middle SDI | 5912050 (5266484 to 6751650) | 1094.89 (975.43 to 1254.57) | 12300862 (11013179 to 13998937) | 942.27 (846.11 to 1065.86) | 108.06% | -13.94% | -0.52 (-0.55 to -0.48) |
| Middle SDI | 10950854 (9944440 to 12298162) | 1221.85 (1103.17 to 1364.82) | 23896857 (21522553 to 26137545) | 960.71 (863.98 to 1047.82) | 118.22% | -21.37% | -0.83 (-0.89 to -0.76) |
| North Africa and Middle East | 3002344 (2668214 to 3383384) | 1940.2 (1715.24 to 2180.55) | 5405417 (4711954 to 6041794) | 1329.39 (1165.64 to 1483.03) | 80.04% | -31.48% | -1.23 (-1.27 to -1.2) |
| Oceania | 22714 (18480 to 28041) | 937.35 (770.88 to 1171.08) | 48691 (40675 to 59299) | 789.38 (663.2 to 979.96) | 114.37% | -15.79% | -0.65 (-0.71 to -0.6) |
| South Asia | 4009489 (3328354 to 5033258) | 809.31 (676 to 1013.15) | 9193297 (8004944 to 11543775) | 690.13 (604.04 to 851.21) | 129.29% | -14.73% | -0.67 (-0.76 to -0.58) |
| Southeast Asia | 3004177 (2687919 to 3310660) | 1355.44 (1203.59 to 1499.12) | 7318328 (6238954 to 8317300) | 1266.45 (1088.13 to 1430.09) | 143.61% | -6.57% | -0.17 (-0.28 to -0.06) |
| Southern Latin America | 424811 (394540 to 451984) | 974.15 (900.63 to 1035.83) | 352925 (323074 to 382286) | 391.27 (357.82 to 424.24) | -16.92% | -59.83% | -2.72 (-2.84 to -2.59) |
| Southern Sub-Saharan Africa | 224900 (194622 to 249787) | 917.26 (782.58 to 1023.35) | 523153 (480110 to 569334) | 1073.71 (979 to 1165.87) | 132.62% | 17.06% | 0.64 (0.22 to 1.06) |
| Tropical Latin America | 1095136 (1036359 to 1137132) | 1368.14 (1271.03 to 1425.63) | 1292888 (1180666 to 1372019) | 520.41 (473.32 to 553.1) | 18.06% | -61.96% | -3.05 (-3.18 to -2.92) |
| Western Europe | 5852489 (5397742 to 6160459) | 963.97 (888.14 to 1017.38) | 3307777 (2903214 to 3626266) | 297.71 (262.76 to 327.93) | -43.48% | -69.12% | -3.95 (-4.1 to -3.79) |
| Western Sub-Saharan Africa | 1138282 (925816 to 1442064) | 1420.18 (1161.28 to 1810.07) | 2185272 (1840340 to 2608225) | 1256.23 (1075.86 to 1478.49) | 91.98% | -11.54% | -0.39 (-0.49 to -0.29) |

EAPC, Estimated Annual Percentage Change; ASR, Age-standardized rate;
