# Supplementary material for: Comprehensive analysis of the ischemic stroke burden at global, regional, and national levels (1990–2021): trends, influencing factors, and future projections
Source: Front Neurol. 2025 Mar 19;16:1492691. doi: 10.3389/fneur.2025.1492691 (PMC11961430; doi:10.3389/fneur.2025.1492691)
Supplement: Supplementary file 4 [file Table_4.DOCX]

Table S4. Attributable age-standardized deaths and DALYs percent by ischemic stroke risk factors in 1990 and by sex in Global

|  | **Age-standardized deaths percent (95% UI)** | | | **Age-standardized DALYs percent (95% UI)** | | |
| --- | --- | --- | --- | --- | --- | --- |
|  | **Overall** | **Male** | **Female** | **Overall** | **Male** | **Female** |
| All risk factor | 88.96 (82.85 to 93.5) | 90.12 (84.73 to 94.17) | 88.06 (81.34 to 93.03) | 88.72 (82.96 to 92.84) | 89.93 (84.72 to 93.53) | 87.62 (81.1 to 92.13) |
|  |  |  |  |  |  |  |
| Level 1 risks |  |  |  |  |  |  |
| Environmental risks | 38.46 (31.7 to 45.08) | 40.49 (33.12 to 47.58) | 36.88 (30.37 to 43.41) | 39.03 (31.98 to 46) | 40.55 (33.02 to 47.9) | 37.65 (31.03 to 44.07) |
| Behavioral risks | 34.16 (22.15 to 45.56) | 44.28 (32.13 to 55.79) | 26.36 (14.34 to 38.48) | 39.73 (28.17 to 49.97) | 49.27 (36.86 to 59.52) | 31.14 (19.86 to 42.76) |
| Metabolic risks | 77.14 (65.92 to 85.88) | 76.21 (65.17 to 85.14) | 77.86 (66.87 to 86.48) | 76.64 (65.23 to 85.19) | 76.12 (65.02 to 84.75) | 77.11 (65.78 to 85.47) |
|  |  |  |  |  |  |  |
| Environmental risks |  |  |  |  |  |  |
| Household air pollution from solid fuels | 15.88 (11.91 to 20.9) | 17.37 (13.14 to 22.5) | 14.73 (11.1 to 19.74) | 31.42 (11.08 to 50.09) | 31.82 (11.66 to 50.08) | 16.98 (13.19 to 22.08) |
| High temperature | 0.5 (-0.02 to 1.31) | 0.58 (-0.01 to 1.48) | 0.43 (-0.04 to 1.18) | 12.98 (9.28 to 17.48) | 13.33 (9.46 to 17.96) | 0.45 (-0.01 to 1.17) |
| Low temperature | 7.35 (6.4 to 8.31) | 7.02 (6.15 to 7.91) | 7.61 (6.6 to 8.61) | 2.53 (1.45 to 3.76) | 2.84 (1.74 to 4.13) | 6.45 (5.6 to 7.33) |
| Lead exposure | 5.64 (-0.74 to 12.56) | 6.94 (-0.91 to 15.47) | 4.63 (-0.6 to 10.32) | 4.75 (1.44 to 8.41) | 4.03 (1.45 to 6.89) | 5.04 (-0.65 to 11.33) |
|  |  |  |  |  |  |  |
| Behavioral risks |  |  |  |  |  |  |
| Smoking | 10.91 (9.06 to 13.07) | 20.24 (17.3 to 23.87) | 3.72 (2.97 to 4.55) | 4.36 (3 to 5.75) | 3.34 (2.3 to 4.44) | 4.67 (3.87 to 5.52) |
| Secondhand smoke | 3.83 (2.61 to 5.13) | 3.03 (2.06 to 4.07) | 4.44 (2.97 to 5.99) | 57.23 (43.56 to 68.28) | 56.16 (42.37 to 67.64) | 5.27 (3.62 to 6.97) |
| Alcohol use | 5.82 (-0.63 to 14.21) | 9.15 (-1.14 to 22.23) | 3.26 (-0.28 to 8.26) | 6.28 (5.47 to 7.09) | 6.1 (5.32 to 6.9) | 3.11 (-0.3 to 7.84) |
| Low physical activity | 3.96 (-0.38 to 8.44) | 3.49 (0.44 to 6.87) | 4.33 (-1.03 to 9.86) | 0.17 (0.08 to 0.26) | 0.15 (0.07 to 0.24) | 5.39 (1.44 to 9.88) |
|  |  |  |  |  |  |  |
| Metabolic risks |  |  |  |  |  |  |
| High systolic blood pressure | 58.94 (44.97 to 70.01) | 57.23 (43.22 to 68.67) | 60.26 (45.95 to 71.31) | 17.63 (13.45 to 22.92) | 18.34 (14.09 to 23.58) | 58.21 (44.33 to 69.23) |
| High LDL cholesterol | 28.24 (9.02 to 47.65) | 28.39 (9.31 to 47.45) | 28.12 (8.78 to 47.78) | 13.8 (11.69 to 15.96) | 23.94 (20.96 to 27.32) | 31.05 (10.76 to 50.1) |
| High fasting plasma glucose | 14.37 (11.32 to 17.73) | 14.84 (11.73 to 18.38) | 14.02 (11.06 to 17.27) | 6.1 (-0.79 to 13.72) | 7.27 (-0.95 to 16.36) | 13.13 (10.39 to 16.21) |
| High body-mass index | 3.8 (0.55 to 7.46) | 3.29 (0.48 to 6.43) | 4.19 (0.6 to 8.25) | 6.01 (-0.76 to 14.67) | 9.23 (-1.33 to 22.26) | 5.13 (0.75 to 9.8) |
| Kidney dysfunction | 9.99 (6.69 to 13.3) | 9.84 (6.85 to 12.9) | 10.1 (6.6 to 13.65) | 0.5 (0 to 1.27) | 0.55 (0.01 to 1.39) | 10.24 (7.23 to 13.37) |
| Diet high in sodium | 8.9 (2.11 to 20.19) | 11.26 (3.2 to 23.42) | 7.07 (1.18 to 17.47) | 0.03 (0.01 to 0.05) | 0.03 (0.01 to 0.05) | 7.82 (1.61 to 18.44) |
| Diet high in red meat | -0.12 (-0.61 to 0.78) | -0.13 (-0.68 to 0.98) | -0.12 (-0.55 to 0.65) | 5.05 (-5.33 to 13.86) | 5.66 (-6.08 to 15.08) | -0.14 (-0.68 to 1.17) |
| Diet low in whole grains | 3.47 (-3.42 to 10.75) | 4.08 (-4.05 to 12.09) | 3 (-2.92 to 9.85) | 13.41 (10.58 to 16.55) | 13.72 (10.84 to 16.98) | 4.5 (-4.65 to 12.67) |
| Diet low in fruits | 2.42 (0.85 to 4.25) | 2.82 (1.32 to 4.73) | 2.11 (0.46 to 3.79) | -0.12 (-0.72 to 1.36) | -0.11 (-0.78 to 1.57) | 3.46 (1.83 to 5.3) |
| Diet low in fiber | 1.2 (-0.07 to 2.9) | 1.38 (-0.06 to 3.13) | 1.07 (-0.12 to 2.68) | 1.1 (0.25 to 1.95) | 0.92 (0.22 to 1.64) | 1.78 (-0.08 to 3.71) |
| Diet low in vegetables | 1.72 (0.53 to 3) | 2.07 (0.83 to 3.48) | 1.44 (0.19 to 2.63) | 3.77 (2.08 to 5.73) | 4.1 (2.33 to 6.04) | 2.25 (1.2 to 3.43) |
| Diet low in polyunsaturated fatty acids | 0.02 (0.01 to 0.04) | 0.02 (0.01 to 0.05) | 0.02 (0.01 to 0.04) | 4.6 (0.68 to 8.75) | 4.02 (0.59 to 7.65) | 0.02 (0.01 to 0.05) |
| Diet high in sugar-sweetened beverages | 0.18 (0.08 to 0.27) | 0.16 (0.08 to 0.24) | 0.19 (0.09 to 0.29) | 9.84 (2.59 to 21.21) | 12.08 (3.73 to 24.29) | 0.18 (0.09 to 0.28) |
| Diet high in processed meat | 1.11 (0.26 to 1.99) | 0.91 (0.22 to 1.63) | 1.26 (0.28 to 2.22) | 10.11 (7.32 to 12.96) | 9.96 (7.41 to 12.7) | 1.26 (0.28 to 2.24) |
